# Laparoscopic Roux-en-Y gastric bypass versus laparoscopic mini gastric bypass in the treatment of obesity: study protocol for a randomized controlled trial

**DOI:** 10.1186/s13063-017-1957-9

**Published:** 2017-05-22

**Authors:** Marko Kraljević, Tarik Delko, Thomas Köstler, Elena Osto, Thomas Lutz, Sarah Thommen, Raoul A. Droeser, Lincoln Rothwell, Daniel Oertli, Urs Zingg

**Affiliations:** 1 Department of General Surgery, University Hospital Basel, 4031 Basel, Switzerland; 2 Department of General Surgery, Limmattal Hospital, 8952 Zurich-Schlieren, Switzerland; 3 IFNH Laboratory of Translational Nutrition Biology, ETH Zurich, 8603 Schwerzenbach, Switzerland; 4 Institute of Veterinary Physiology, Vetsuisse Faculty and Centre of Integrative Human Physiology, University of Zurich, 8057 Zurich, Switzerland; 5 Basel Institute for Clinical Epidemiology and Biostatistics, University Hospital Basel, 4031 Basel, Switzerland; 6 Department of General Surgery, Ipswich General Hospital, Ipswich, Queensland 4305 Australia

**Keywords:** Randomized controlled trial, Roux-en-Y gastric bypass, Mini gastric bypass, Outcome, Excess weight loss

## Abstract

**Background:**

Laparoscopic Roux-en-Y gastric bypass (LRYGB) is considered the gold standard in bariatric surgery, achieving durable long-term weight loss with improvement of obesity-related comorbidities. Lately, the laparoscopic mini gastric bypass (LMGB) has gained worldwide popularity with similar results to LRYGB in terms of weight loss and comorbidity resolution. However, there is a lack of randomized controlled trials (RCT) comparing LMGB and LRYGB. This article describes the design and protocol of a randomized controlled trial comparing the outcomes of these two bariatric procedures.

**Methods/Design:**

The trial is designed as a single center, randomized, patient and observer blinded trial. The relevant ethics committee has approved the trial protocol. To demonstrate that LMGB is not inferior to LRYGB in terms of excess weight loss (EWL) the study is conducted as a non-inferiority trial with the sample-size calculations performed accordingly. EWL 12 months after surgery is the primary endpoint, whereas 3-year EWL, morbidity, mortality, remission of obesity related comorbidities, quality of life (QOL) and hormonal and lipid profile changes are secondary endpoints. Eighty patients, 18 years or older and with a body mass index (BMI) between 35 and 50 kg/m^2^ who meet the Swiss guidelines for the surgical treatment of morbid obesity will be randomized. The endpoints and baseline measurements will be assessed pre-surgery, peri-surgery and post-surgery (fixed follow up measurements are at discharge and at the time points 6 weeks and 12 and 36 months postoperatively).

**Discussion:**

With its 3-year follow up time, this RCT will provide important data on the impact of LMGB and LRYGB on EWL, remission of comorbidities, QOL and hormonal and lipid profile changes.

**Trial registration:**

ClinicalTrials.gov, NCT02601092. Registered on 28 September 2015.

**Electronic supplementary material:**

The online version of this article (doi:10.1186/s13063-017-1957-9) contains supplementary material, which is available to authorized users.

## Background

The globally growing incidence of obesity and its related comorbidities is one of the most challenging public health issues [1]. Morbid obesity reduces life expectancy, in particular among younger adults [2]. Bariatric surgery, the only effective treatment for morbidly obese patients, has shown effective long-term weight loss and significant reduction of obesity-related comorbidities and mortality in randomized controlled trials (RCT) [3–5]. The laparoscopic Roux-en-Y gastric bypass (LRYGB) is considered the gold standard among the different bariatric procedures, achieves durable long-term weight loss and improves obesity-related comorbidities [6, 7]. The laparoscopic mini gastric bypass (LMGB), first reported by Rutledge et al. [8], has recently gained popularity as a new procedure for the treatment of morbid obesity. LMGB is suggested to be a technically less demanding operation compared to LRYGB and offers comparable benefits. Several studies have been published assessing the efficacy and safety of LMGB. The first and only randomized controlled trial comparing the LRYGB to LMGB was carried out by Lee et al. in 2005 [9]. The authors observed excess weight loss (EWL) of 64.9% after LMGB and of 64.4% after one and two years, respectively. In comparison, patients with LRYGB had EWL of 58.7% and 60.0%, respectively. Excess weight (EW) is defined as the amount of weight that is in excess of the ideal body weight (IBW), which is determined as a body mass index (BMI) of 25 kg/m^2^. Therefore, EWL is the quotient of weight loss and EW. In addition, LMGB patients had fewer postoperative complications and shorter hospitalization.

Given the increasing number of bariatric procedures performed worldwide [10], the impact on weight loss, morbidity and mortality of LMGB in comparison to LRYGB needs to be further assessed in randomized controlled trials. Therefore, the aim of the current trial is to analyze the EWL and resolution of comorbidities with LMGB and LRYGB. Furthermore, the differences in hormone levels and changes in the lipid status of patients undergoing one of these two operations will be analyzed. These results have the potential to influence bariatric surgery guidelines profoundly and may bring additional insights in the pathophysiology of the intestinal tract after bariatric surgery.

## Methods/Design

### Primary objective

The general objective of this study is to compare LMGB to LRYGB in terms of clinical outcome and safety parameters. The primary objective of the study is to evaluate the non-inferiority of LMGB in comparison to LRYGB in terms of EWL (percent) one year after surgery.

### Secondary objective

The secondary objectives are the long-term effects of LMGB on weight loss, the perioperative and postoperative morbidity and the changes in different pathophysiological parameters during the study period: EWL at 36 months, morbidity, mortality, operative time, length of stay, subjective perception of appetite and satiety, quality of life (QOL), incidence of dumping syndrome, hormone levels (ghrelin, glucagon-like peptide 1, peptide YY, insulin), bile acids and lipid profile and functionality.

### Study design and site

**Fig. 1:**
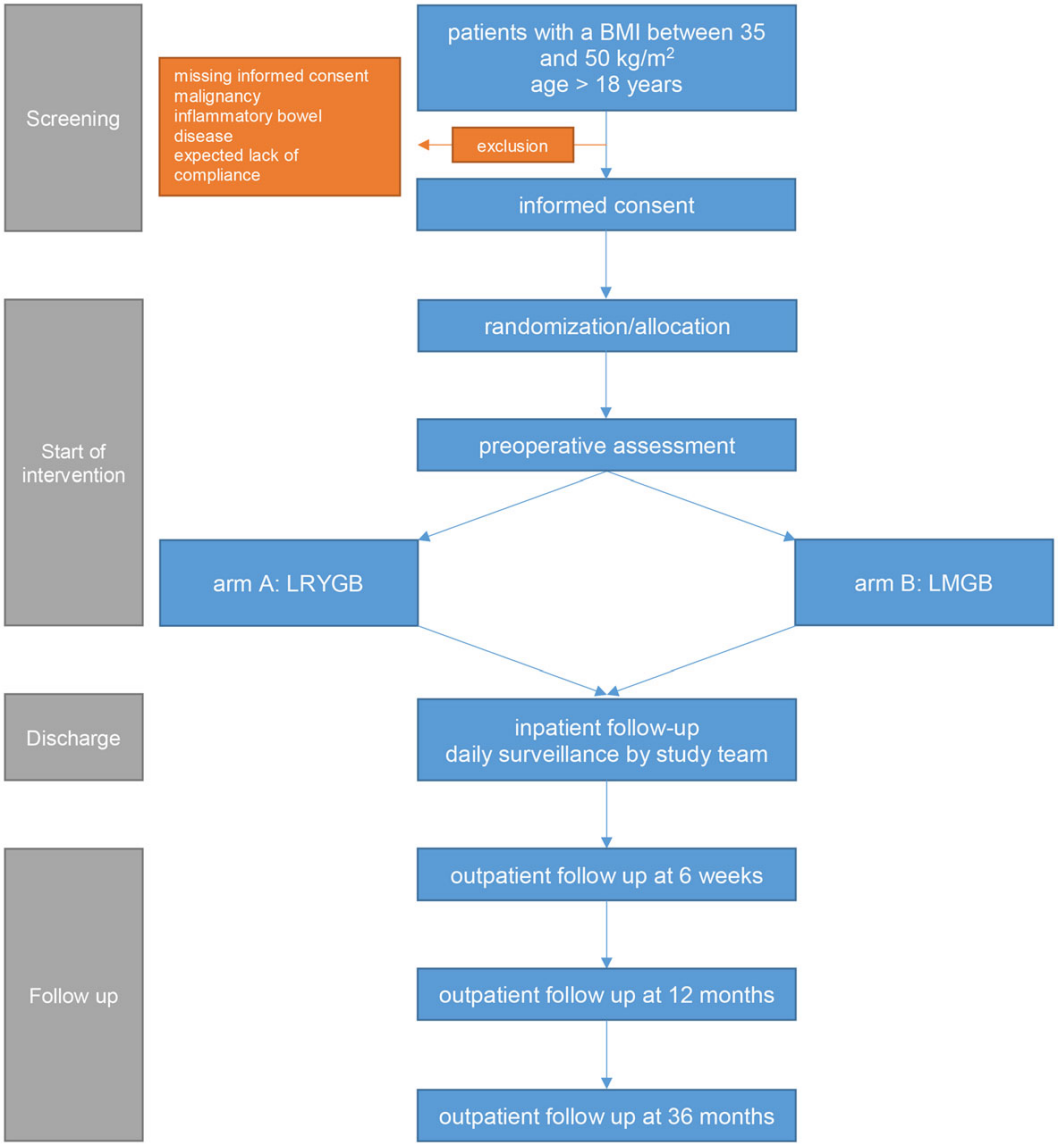
Schedule of enrollment, interventions, and assessments according to the Standard Protocol Items: Recommendations for Interventional Trials (SPIRIT) guideline. *LRYGB* laparoscopic Roux-en-Y gastric bypass, *LMGB* laparoscopic mini gastric bypass, *GERD* gastroesophageal reflux disease, *QOL* quality of life

This single center, randomized, controlled, patient and observer blinded, non-inferiority trial is being conducted at the Limmattal Hospital in Zurich-Schlieren, a reference center for bariatric surgery according to the Swiss Study Group for Morbid Obesity (SMOB). The trial has been registered on ClinicalTrials.gov under the identifier NCT02601092. This protocol has been written in accordance with the Standard Protocol Items: Recommendations for Interventional Trials (SPIRIT) guidelines (Additional file 1). A figure showing the planned visit and examination schedule is presented in Fig. 1.

### Sample size

A total of 80 patients will be randomized, with 40 patients assigned to each treatment arm. A loss of follow up at one year of 2.5% is considered realistic and it has been calculated to account for a dropout rate of 10%.

### Inclusion criteria

Patients aged 18 years or older with a BMI between 35 and 50 kg/m^2^ and eligible for bariatric surgery according to the SMOB guidelines will be enrolled.

### Exclusion criteria

Patients will be excluded on the following basis:

- Missing informed consent
- Lack of conservative weight loss treatment over 2 years
- Malignancy
- Inflammatory bowel disease
- Expected lack of compliance after multidisciplinary evaluation for surgery
- Pregnancy
- BMI over 50 kg/m^2^

Preoperatively, patients will be assessed by a nutritionist, endocrinologist, psychiatrist and general surgeon. If more than three appointments are not attended or if there are any contraindications due to severe psychiatric comorbidities, the operation will be postponed or cancelled.

### Randomization

Eligible patients, who give confirmed consent, are randomly assigned to one of the two operating techniques. Randomization will be performed using sequentially numbered, sealed, opaque envelopes. Computer-generated block randomization is used to ensure an equal number of patients in each treatment group. The block size used is not available from the study protocol and is unknown to the investigator, to keep the potential for selection bias at the lowest level possible. Patients will be allocated to a study group by the operating surgeon immediately prior to the operation.

### Interventions

**Fig. 2:**
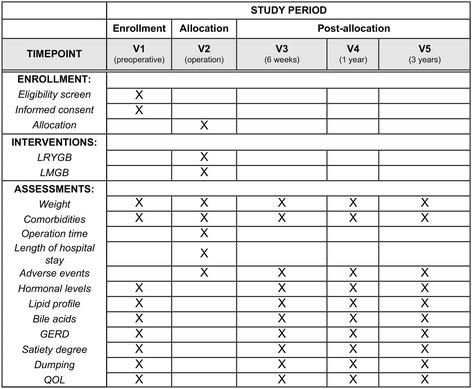
Flow diagram of the study design. *LMGB* laparoscopic mini gastric bypass, *LRYGB* laparoscopic Roux-en-Y gastric bypass, *BMI* body mass index

Patients will receive standard preoperative assessment including endocrine, pulmonary function and cardiovascular assessment, psychological assessment, gastroscopy with *Helicobacter pylori* testing and abdominal ultrasound to check for liver size and gallstones. The operation will always be performed by the same surgeon with experience of performing over 1000 bariatric procedures. Blinded preoperative and postoperative follow up will be independently performed by two study physicians. The study design flow diagram is presented in Fig. 2.

### Laparoscopic Roux-en-Y gastric bypass (LRYGB)

**Fig. 3:**
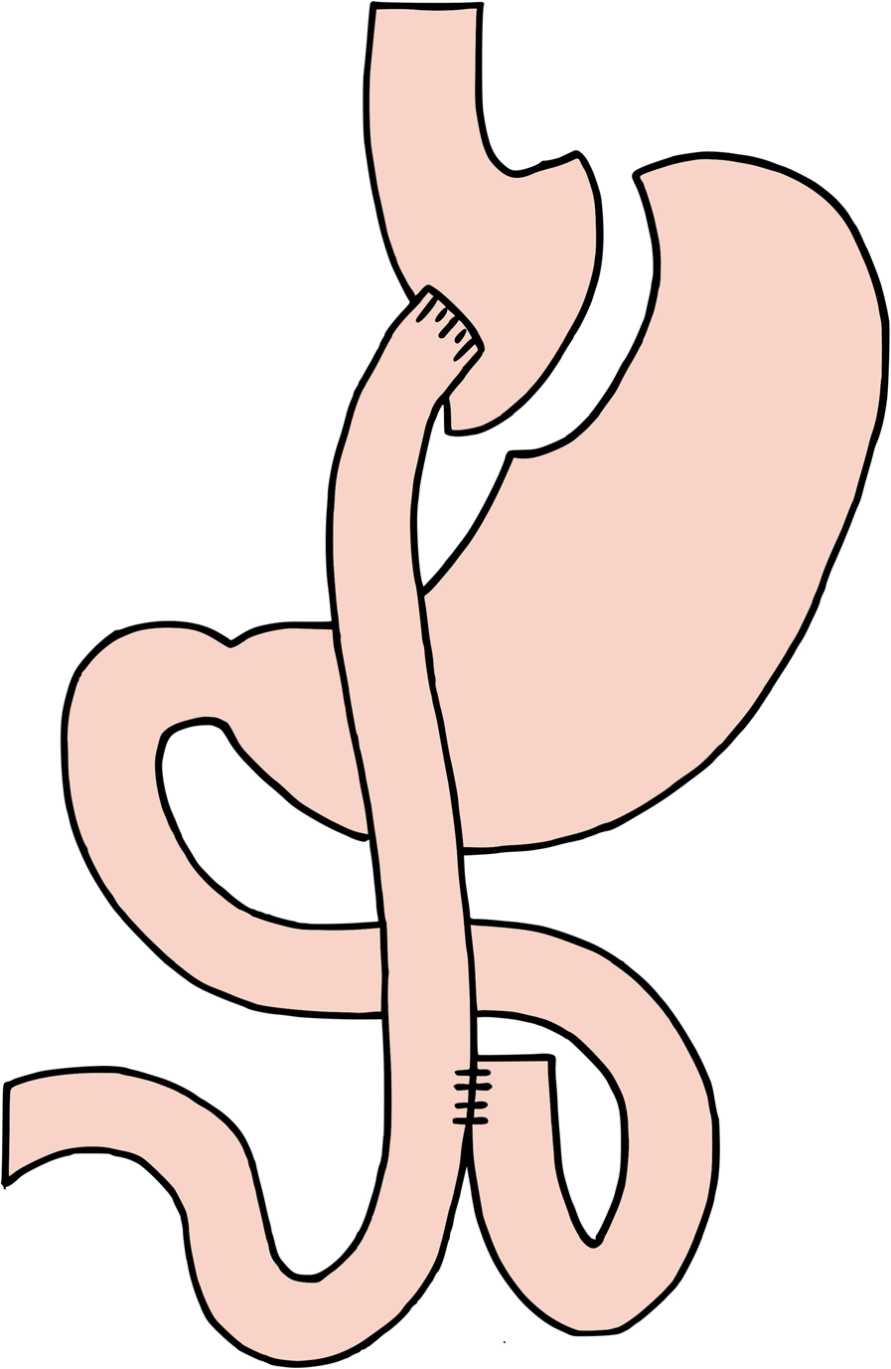
Laparoscopic Roux-en-Y gastric bypass. The alimentary limb is 150 cm in *length* and is in the antecolic position. The mesentery defect is closed with interrupted sutures

After gastroesophageal junction identification, the stomach is transected with a linear stapler entering the lesser sac 3–5 cm below the junction, creating a small gastric pouch with a volume of approximately 30 ml. A 60-cm biliopancreatic limb is measured and an antecolic end-to-side gastroenterostomy is formed using a 25-mm circular stapler. A stapled side-to-side jejunojejunostomy is formed 150 cm distally (Fig. 3). Intermesenteric and Petersen spaces are closed with non-absorbable sutures. All patients will be discharged on daily multivitamin and calcium tablets. Vitamin levels will be assessed during follow up and supplementation undertaken if required.

### Laparoscopic mini gastric bypass (LMGB)

**Fig. 4:**
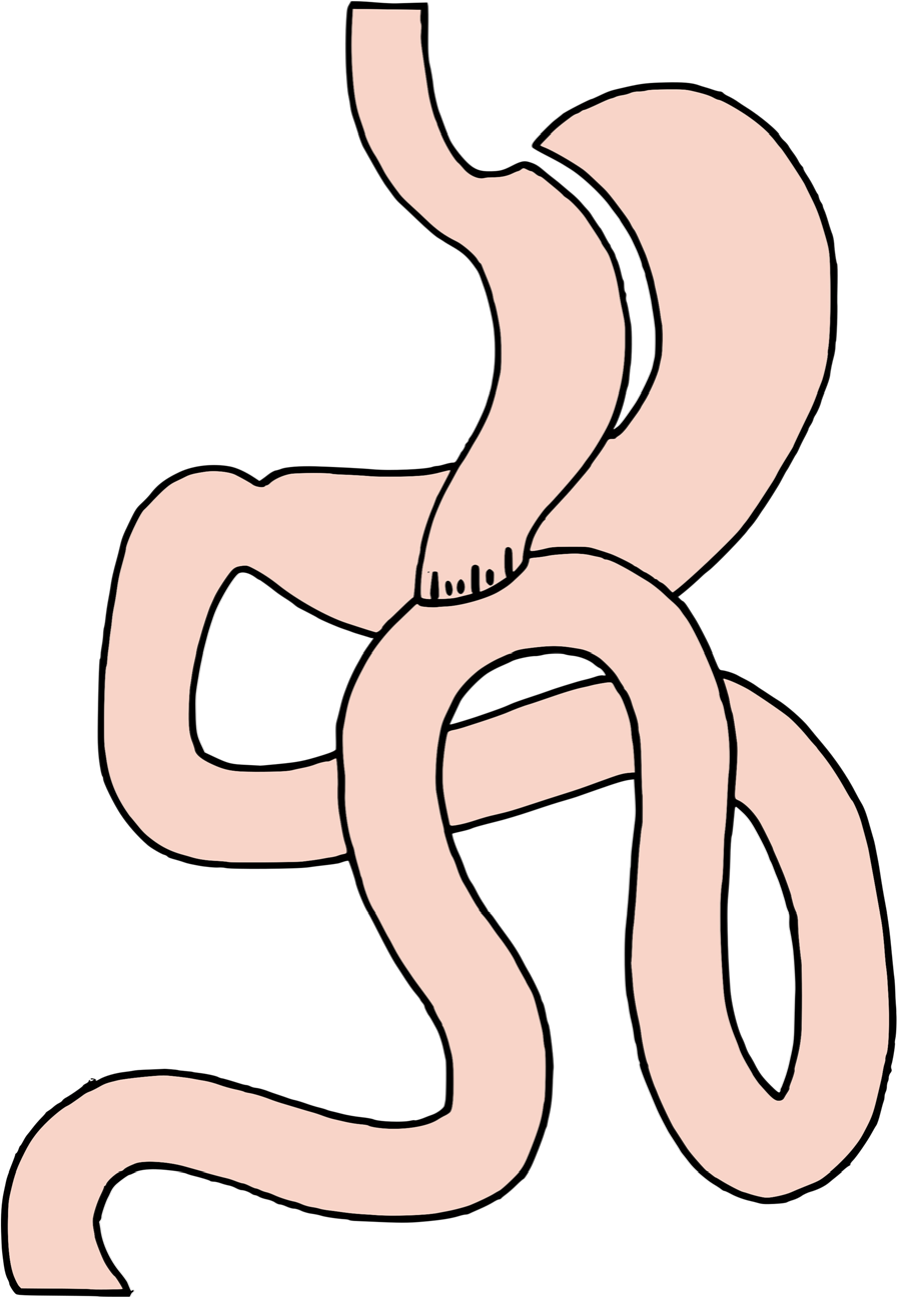
Laparoscopic mini gastric bypass. The *narrow* gastric tube is the *diameter* of a 36 French bougie. The gastroenterostomy is created at the small bowel 200 cm distal to the Trietz ligament

Stapled division of the stomach at the junction of the body and antrum is undertaken, at a location where a jejunal loop can be comfortably brought up (Fig. 4). A 36 French bougie is passed by the anesthetist and held against the lesser curvature. A very long gastric pouch is created by resection of the stomach parallel to the lesser curvature towards the angle of His. No short gastric vessels are divided. The bypassed stomach lies on the patients left, and the narrow lesser-curvature gastric pouch lies on the patient’s midline to the right of the bypassed stomach. A point is selected on the small bowel 200 cm distal to the ligament of Treitz. The jejunal loop is brought up lying antecolic, and a linear stapler (60 mm) is used to join the stomach and the small bowel at this point. The linear stapler enterotomy site is then closed with a running suture. The greater omentum is tucked between the gastric tube and the bypassed stomach [8]. Finally, Petersen’s space is closed with a non-absorbable suture. All patients will be discharged with daily oral multivitamins and calcium tablets. Vitamin levels will be assessed regularly during follow up and supplementation undertaken if required.

### Study visits

Physicians and study nurses blinded to the intervention will perform study documentation and patient assessment. Since the trial is designed as an observer and patient blinded RCT, information about the surgical procedure will not be disclosed to any assessors during the follow up examinations. There will be five study visits in total. The first visit will be preoperative after informed consent is obtained. Postoperatively, study visits will be performed at discharge, 6 weeks and 12 and 36 months. Each study visit includes collection of data on weight, blood tests, in particular, metabolic parameters such as hormonal assessment, glucose levels and lipid profile. Measurement of metabolic parameters will be conducted preoperatively and 6 weeks and 12 and 36 months postoperatively after a 12-hour fast and at 60 and 120 minutes after a standardized test meal (375 kcal). In addition, questionnaires will be also assessed (Gastrointestinal Quality of Life Index (GIQLI), Simplified Nutritional Appetite Questionnaire (SNAQ), Gastroesophageal Reflux Disease Questionnaire (GERDQ), Bariatric Analysis And Reporting Outcome System (BAROS) and the Sigstad score) at each visit. Furthermore, triglycerides (TG), total cholesterol, high-density lipoprotein (HDL), low-density lipoprotein (LDL), ghrelin, glucagon-like peptide-1 (GLP-1), peptide YY (PYY), glucose, insulin and bile acids will also be evaluated. Postoperatively additional data on morbidity (surgical and non-surgical short-term and long-term complications) and mortality will be collected.

### Study endpoints

#### Primary endpoint

The primary endpoint is the EWL at 12 months after surgery (LRYGB or LMGB). For the EWL calculation (percent) the pre-surgical total body weight (at the patient’s hospital entry time) and postsurgical total body weight at one year postoperative is measured.

#### Secondary endpoints

- Long-term EWL at 36 months after surgery (in percent)
- Early surgical and non-surgical complications (≤30 days) according to the Clavien-Dindo classification [11]
- Operation time
- Length of hospital stay
- Hormonal levels (ghrelin, GLP-1, PYY, glucose and insulin)
- Lipid profile
- Bile acids
- Incidence of GERD
- Satiety degree
- Dumping
- Quality of life questionnaires

Levels of glucose, insulin and HbA1c will be determined for assessment of glucose homeostasis. TG, total cholesterol, LDL cholesterol (LDL-C), HDL cholesterol (HDL-C) and HDL/LDL cholesterol quotients will be measured to create a lipid profile. Quality of life, satiety degree, incidence of reflux and dumping will be investigated by questionnaires (GIQLI [12], SNAQ [13], GERDQ [14], BAROS [15] and the Sigstad score [16]). Secondary endpoints will be evaluated preoperatively, at discharge, 6 weeks and 12 and 36 months postoperatively.

### Blinding

With the exception of the team in the operating theatre, all medical and non-medical practitioners and patients will be blinded to the procedure. The procedure will be named as *study bypass* in all medical records. Unblinding is permitted in the case of surgical or medical complications, emergency consultation or other ethical considerations.

### Statistical analyses

#### Sample size

If LMGB is non-inferior to LRYGB, 72 patients are required (36 patients per study arm) to have an 80% chance that the lower limit of the one-sided 97.5% confidence interval (equivalently a 95% two-sided confidence interval), does not include the non-inferiority limit of –10% EWL when assessed at 12 months. Given an anticipated dropout rate of 10%, the total number of patients to be included in the study is 80 (40 patients per arm). The non-inferiority limit of –10% EWL was chosen based on expert bariatric opinion. Furthermore a standard deviation of 15 was assumed in both arms according to a study from Lee et al. [9]. For further details on calculations see “Analysis of endpoints”.

#### Analysis of endpoints

The study results will be reported in adherence to the extension of the Consolidated Standards of Reporting Trials (CONSORT) statement from 2010 on reporting of non-inferiority randomized trials [17]. Summary statistics will be used to describe and compare patient characteristics of all suitable but non-included patients and all included patients (overall and stratified for the two treatment arms). Study endpoints will primarily be analyzed for the per protocol (PP) population. Sensitivity analysis will be conducted for the intention-to-treat (ITT) population. Thereby the ITT population includes all randomized patients in the groups to which they were randomly assigned, regardless of their adherence to the entry criteria, regardless of the treatment they actually received and regardless of subsequent withdrawal or deviation from the protocol. In the PP population all protocol violators, including anyone who switched groups or missed measurements are excluded. For each group, number of participants (denominator) included in each analysis and whether the analysis was by original assigned groups will be given. Additional sensitivity analysis will be used if, despite all efforts taken to ensure complete data collection, the number of missing data is non-negligible or could potentially bias the results and conclusions.

#### Analysis of primary endpoint

The primary endpoint is the EWL at 12 months post-surgery. To examine non-inferiority of the experimental compared to standard treatment, we assess if the two-sided confidence interval of the difference between the mean EWL in the experimental treatment group and the mean EWL in the standard treatment group does not include the non-inferiority limit of –10% EWL.

#### Analysis of secondary endpoints

To investigate non-inferiority of the experimental treatment in the long term, analysis used for the primary endpoint will be repeated for EWL at 36 months post-surgery. All other endpoints will be analyzed under the assumption of superiority of the experimental treatment compared to the standard treatment. To compare the duration of surgery and the length of hospital stay between the two treatment groups, the Wilcoxon signed-rank test will be conducted (as we assume the data to be right-skewed).

The number of perioperative and postoperative complications will be analyzed by the chi-squared test or Fisher’s exact test, dependent on the observed numbers in a 2 × 2 contingency table. Additionally, we will compare the risk for complications in the two groups by calculating the risk difference with 95% confidence interval. The other secondary endpoints will be investigated using appropriate explorative methods and graphical visualization.

### Ethical considerations

Participation in this trial is strictly voluntary and patients are allowed to exit the trial at any point without explanation. All eligible patients are provided an information sheet describing the study with sufficient information for them to make an informed decision about their participation in this study.

The study protocol, patients’ information sheets and informed consent forms were approved by the local ethics committee (Kantonale Ethikkommission Zürich, KEK-ZH 2013-0389). In addition, insurance coverage for general liability has been obtained. Patients who decline to participate in this study are treated according to clinical standards. These patients will not be included and no study-specific follow up will be performed.

### Participants’ confidentiality

The participants’ confidentiality is maintained at all times. For confidentiality reasons, case report forms (CRF) do not contain any personal data on study participants. Members of the ethics committees are obliged to respect confidentiality and to refrain from divulging the participants’ identities or any other personal information they might be aware of. Source data in the hospital’s electronic patient information systems are secured by personal passwords and handled with respect to medical secrecy.

### Archiving and data retention

The investigator will maintain all study-related records, such as CRFs, medical records, laboratory reports, informed consent documents, safety reports, information regarding participants who discontinued the trial and other pertinent data. All records will be retained by the investigator as long as required by the applicable laws and regulatory requirements (10 years). Thereafter, all data will be destroyed. The study is conducted in compliance with this protocol and according to Good Clinical Practice standards and legal regulations. Serum and plasma samples will be stored in –80 °C freezers located in the laboratories where the measurements will be performed, which are the Center for Molecular Cardiology, Institute of Veterinary Physiology at the University of Zurich and the Laboratory of Translational Nutrition Biology at the Federal Institute of Technology (ETH) Zurich.

Freezers are provided with systems for 24-hour monitoring 7 days a week and reporting of temperature control with immediate remote alarm notification and continuous data collection. This provides reliable and durable environmental conditions for temperature requirements of our samples. The serum and plasma samples will be stored for up to 5 years after the completion or termination of the study. After this period, samples will be disposed of as biohazardous waste following policies in force at the University of Zurich and at ETH Zurich for the handling and disposal of biohazardous material. At the completion of the study, there will be a final reconciliation of samples collected, samples used and samples remaining. This reconciliation will be reported on a samples accountability form, signed and dated. All stored samples will be coded with a unique storage identifier. The list of patient identifiers is accessible to UZ, TD, MK and EO. The scientists who will carry out analyses on these materials are all experienced researchers. They will not have access to personal identifiers and will not be able to link the results of these tests to personal identifier information. Access to the study results will be restricted. A password system will be utilized to control access. These passwords will be changed on a regular basis. All reports prepared within this study will be prepared such that no individual subject can be identified.

## Discussion

The positive effects of bariatric surgery on weight loss and obesity-related comorbidities and mortality have been widely demonstrated in long-term cohort trials and short-term RCTs [6, 7, 18]. Over time, these procedures have improved in respect to safety and can be offered at a low mortality and morbidity rate [9, 19]. Various surgical options are available, with LRYGB and laparoscopic sleeve gastrectomy (LSG) being the two most common procedures. In addition, there are many new operations, one of them being the LMGB. The quest for the most effective bariatric procedure is not yet completed as weight loss and improvement of metabolic status, comorbidity and quality of life have only been evaluated in a few randomized controlled trials over a short-term period. This study investigates the effectiveness of LMGB compared to LRYGB, analyzing defined clinical endpoints such as EWL, morbidity and mortality, metabolic changes and quality of life. In addition, the RCT will also answer some important questions about LMGB, such as the course of obesity-related comorbidities and dumping syndrome.

Furthermore, this study addresses the postoperative course of hormones and lipids in these two different bariatric procedures. It has been shown that after bariatric operations the levels of hormones such as GLP-1 and PYY are markedly increased [20]. Surgery improves obesity-associated pro-atherogenic dyslipidemia as well, which is characterized by high LDL and TG and low HDL-C plasma levels. Low HDL-C levels are associated with increased cardiovascular disease risk [21]. It was recently demonstrated that RYGB improved the endothelial protective properties of HDL in obese patients; 12 weeks after RYGB these properties were comparable to those of healthy subjects although the patients were still obese [22, 23]. Overall, the data indicate that RYGB may achieve a dual HDL benefit, first the rapid restoration of quality to more “healthy” HDL, and second the increase in circulating HDL levels in the longer-term period. The influence of LMGB on changes in lipoprotein profile and function and bile acids are not yet known.

One issue surrounding LMGB that remains highly controversial is the reflux of bile into the gastric pouch and potentially into the esophagus. LMGB is often compared to the refluxogenic loop gastric bypass (LGB) described by Mason et al. [24], which consists of a large gastric pouch transected horizontally with the gastrojejunostomy close to the gastroesophageal junction. However, LMGB consists of a long narrow vertical gastric pouch, thus, reflux into the esophagus is not believed to be an LMGB-specific problem. By contrast, the surgical technique of LMBG is comparable to the Billroth II procedure performed in peptic ulcer disease and gastric cancer. In LMBG and Billroth II, the most important parallel is the single anastomosis bypass of the duodenum without a Roux-limb. The LMGB distance of the gastrojejunostomy to the esophagus is longer than in Billroth II. Furthermore, the biliopancreatic limb in LMGB measures 200 cm compared to 60–80 cm for the Billroth II operation.

In conclusion, the perfect bariatric procedure is technically easy and safe, and leads to adequate EWL and improvement in obesity-related comorbidities. In contrast to the widely used LRYGB and LSG, the LMGB might fulfill all of these criteria. The current study will answer questions about safety, effectiveness and pathophysiological changes in obesity-relevant hormones and lipids after LMGB. These findings might therefore influence decision-making in bariatric surgery implementing current surgical protocols towards personalized indications.

## Trial status

The trial has received ethics approval, and enrollment in the trial has begun but has not yet reached full enrollment.

## Additional file

Additional file 1: SPIRIT checklist of included items in our clinical trial protocol (DOCX 64 kb)

## Abbreviations

BAROS: Bariatric Analysis and Reporting Outcome System
BMI: Body mass index
CRF: Case report form
EGD: Esophagogastroduodenoscopy
EW: Excess weight
EWL: Excess weight loss
GERDQ: Gastroesophageal Reflux Disease Questionnaire
GIQLI: Gastrointestinal Quality of Life Index
GLP-1: Glucagon-like peptide 1
HDL: High density lipoprotein
IBW: Ideal body weight
ITT: Intention-to-treat
LDL: Low density lipoprotein
LMGB: Laparoscopic mini gastric bypass
LRYGB: Laparoscopic Roux-en-Y gastric bypass
PP: Per protocol
PYY: Peptide YY
QOL: Quality of life
RCT: Randomized controlled trial
RYGB: Roux-en-Y gastric bypass
SMOB: Swiss Study Group for Morbid Obesity
SNAQ: Simplified Nutritional Appetite Questionnaire
SPIRIT: Standard Protocol Items: Recommendations for Interventional Trials
TG: Triglycerides
